# Temporal concordance between pulse contour analysis, bioreactance and carotid doppler during rapid preload changes

**DOI:** 10.1371/journal.pone.0265711

**Published:** 2022-03-23

**Authors:** Jon-Émile S. Kenny, Igor Barjaktarevic, Andrew M. Eibl, Matthew Parrotta, Bradley F. Long, Mai Elfarnawany, Joseph K. Eibl

**Affiliations:** 1 Health Sciences North Research Institute, Sudbury, ON, Canada; 2 Division of Pulmonary and Critical Care, Department of Medicine, David Geffen School of Medicine at UCLA, Los Angeles, CA, United States of America; 3 Northern Ontario School of Medicine, Sudbury, ON, Canada; Universita degli Studi Magna Graecia di Catanzaro, ITALY

## Abstract

**Purpose:**

We describe the temporal concordance of 3 hemodynamic monitors.

**Materials and methods:**

Healthy volunteers performed preload changes while simultaneously wearing a non-invasive, pulse-contour stroke volume (SV) monitor, a bioreactance SV monitor and a wireless, wearable Doppler ultrasound patch over the common carotid artery. The sensitivity and specificity for detecting preload change over 3 temporal windows (early, middle and late) was assessed.

**Results:**

40 preload changes were recorded in total (20 increase, 20 decrease). Immediately, the wearable Doppler had high sensitivity (100%) and specificity (100%) for detecting preload change with an area under the receiver operator curve (AUROC) of 0.98 for both velocity time integral (VTI, 10.5% threshold) and corrected flow time (FTc, 2.5% threshold). The sensitivity, specificity and AUROC for non-invasive pulse contour were equally good (9% SV threshold). For bioreactance, a 13% SV threshold immediately detected preload change with a sensitivity, specificity and AUROC of 60%, 95% and 0.75, respectively. After two SV outputs following preload change, the sensitivity, specificity and AUROC of bioreactance improved to 70%, 90% and 0.85, respectively.

**Conclusions:**

Carotid Doppler ultrasound and non-invasive pulse contour detected rapid hemodynamic change with equal accuracy; bioreactance improved over time. Algorithm-lag should be considered when interpreting clinical studies.

## Introduction

Assessment of stroke volume (SV) after altered cardiac preload expresses the functional state of the heart [1]. Normally, SV rises following increased cardiac preload. By contrast, little SV augmentation implies abnormal cardiac function, for example, a heart no longer responsive to clinical preload expansion such as intravenous fluids [2].

Given the above, tracking rapid SV change at the bedside is clinically significant. Accordingly, Doppler of the common carotid artery has been employed as a surrogate for SV [3–7]. In healthy volunteers, we have shown perfect directional concordance between non-invasive pulse contour analysis and carotid Doppler in over 100 preload changes measured by a wireless, wearable ultrasound [8, 9]. As well, in a human model of hemorrhage and resuscitation, we noted a strong, linear association between changing SV and carotid artery Doppler [10]. Yet, in a feasibility study comparing Doppler of the carotid artery, descending aorta, non-invasive pulse contour analysis and bioreactance, we noted clinically-relevant temporal discordance despite directional concordance [11]. In other words, with a rapid increase in cardiac preload, carotid and descending aortic velocity time integral (VTI) rose abruptly, whereas SV by bioreactance only did so after a time-delay. Conversely, on rapid preload reduction, the fall in carotid and aortic VTI was instant while bioreactance SV lagged. Clarifying temporal concordance between changing physiology and the response recorded by a monitoring device is critical as timing mismatch may cause false positives and negatives and, potentially, lead to clinical mismanagement.

To describe the temporal relationship between a wearable carotid Doppler, non-invasive pulse contour and bioreactance SV monitors, we studied healthy volunteers simultaneously wearing all 3 devices while performing rapid preload modifying maneuvers. We separated each preload change into early, middle and late temporal windows. Because the wearable Doppler has the resolution of a single cardiac cycle, we hypothesized that it would have better sensitivity and specificity for detecting preload change across all temporal assessment windows whereas non-invasive pulse contour analysis and bioreactance would have rising AUROC over time because of data-smoothing and/or algorithm-lag.

## Materials and methods

### Clinical setting

We studied 8 healthy volunteers with no known cardiovascular history and on no regular cardiovascular medications. The procedures followed were in accord with the ethical standards of the committee on human experimentation at our institution. Written and informed consent was obtained for all subjects, and the study was approved by the Research Ethics Board of Health Sciences North.

### Stand-squat-stand paradigm

As previously described [9] resting stand, squat and return-to-stand were each 72 seconds in duration. The reason for this duration is because the bioreactance monitor updates stroke volume every 24 seconds, while the non-invasive pulse contour analysis device gives SV every 20 seconds. Thus, we were able to capture 3 SV values during each preload change for both devices. Heart rate and mean arterial pressure (MAP) were recorded by the non-invasive pulse contour device. To minimize motion artifact in all 3 devices, the subjects practiced the squat and stand prior to recording and were instructed to rigidly maintain head, arm and thoracic spine positioning throughout.

### Stroke volume monitoring

The Clearsight® (Edwards Lifesciences, Irvine, California) was applied to the third digit of each subject in the standing position. The protocol did not begin until there was adequate Clearsight® signal as measured by the Physiocal metric (i.e. ≥ 50). Each subject kept their arm fully relaxed and extended throughout the protocol. Though hemodynamic data, including SV, were assessed continuously across the first 72 seconds of quiet standing, we chose the final full SV value immediately before squat as baseline for increased preload (↑ preload). On squat, we chose the first 3 SV values as early (↑ preload_early_), middle (↑ preload_middle_) and late (↑ preload_late_), respectively. Similarly, on return-to-stand (↓ preload), the first 3 SV values were analyzed as ↓ preload_early_, ↓ preload_middle_ and ↓ preload_late_, respectively, and compared to the final SV value before return-to-stand as baseline. An identical approach was used for the bioreactance device (Cheetah NICOM, Baxter Medical, Deerfield, Illinois), though SV values were updated every 24 seconds. Data was extracted from both the pulse contour and bioreactance devices via USB into autogenerated spreadsheets provided by the manufacturer. Time-stamps on the spreadsheets were used to synchronize the devices.

### Corrected carotid flow time and VTI monitoring

**Fig 1:**
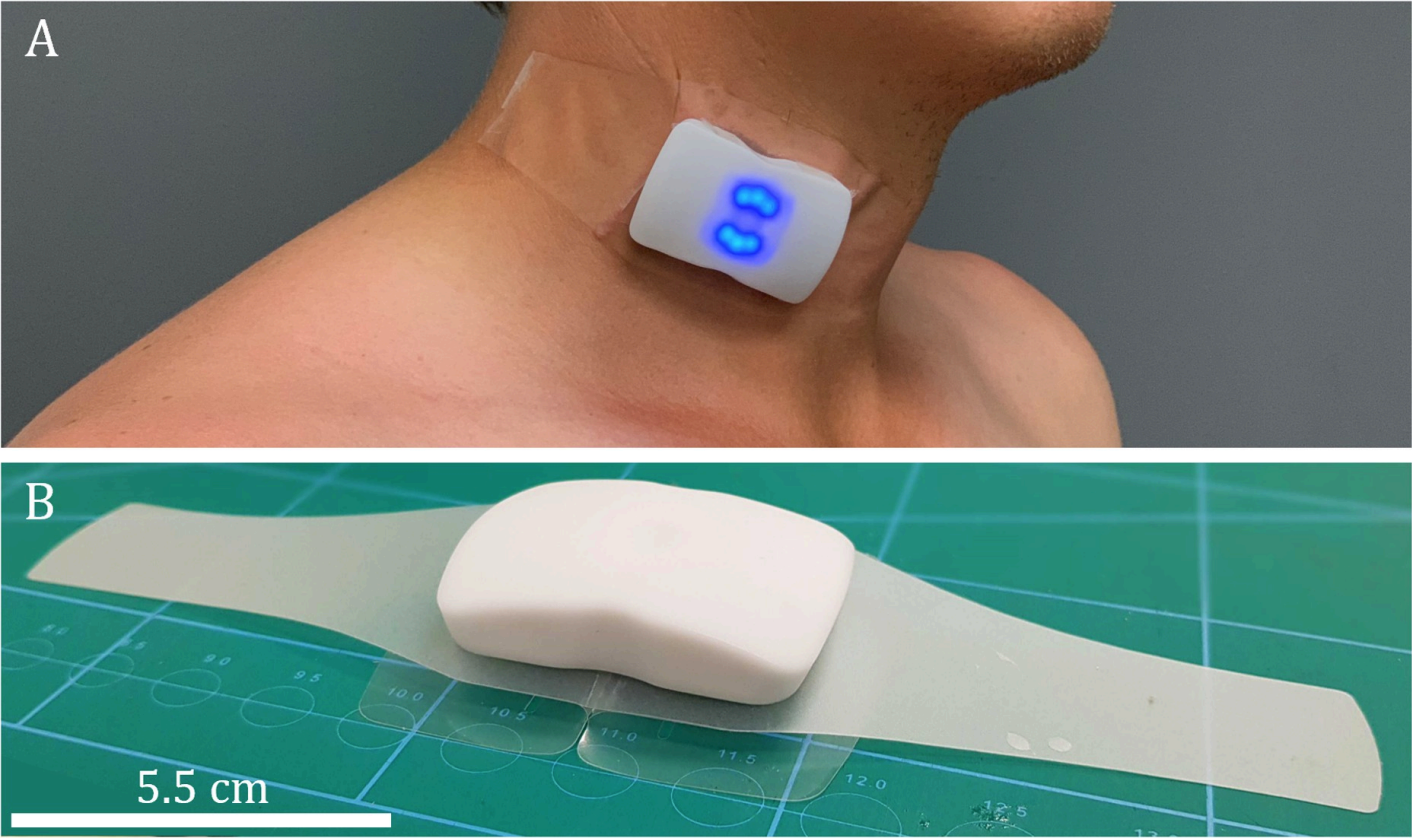
A and B: The wireless Doppler ultrasound patch.

The U.S. Food and Drug Administration-cleared carotid ultrasound patch (Flosonics Medical, Sudbury, ON.) (Fig 1) was placed by palpation over the carotid artery below the angle of the jaw to ensure Doppler sampling below the carotid artery bifurcation. The maximum velocity of the continuous wave (CW) Doppler pulse was automatically traced using an algorithm based on the approach described by Li et al [12]. The automated maximum velocity estimation for each timepoint in the waveform was used to calculate the VTI as the area under the curve. The wearable Doppler maximum velocity trace has excellent accuracy at tracking change both *in vitro* and *in vivo* [13]. The duration of systole (i.e. from systolic velocity upstroke to the dicrotic notch, in milliseconds) was recorded from the CW Doppler patch and corrected for heart rate using Wodey’s Formula [3] to obtain the corrected carotid flowtime (FTc) in milliseconds (ms):

$$\text{FTc}=\text{systolic}\;\text{flow}\;\text{time}+\left[1.29\;\text{x}\;\left(\text{HR}-60\right)\right].$$ (1)

Data from the carotid Doppler were averaged over 24 second time intervals to match the bioreactance device. The change in carotid Doppler VTI and FTc were analyzed, temporally, in exactly the same way as SV, described above.

### Statistical and temporal concordance analyses

All hemodynamic variables were tested for normality using Kolmogorov-Smirnov analysis and compared using a two-tailed student t-test. Rather than choosing one device as the ‘gold-standard,’ we chose changing physiology (i.e., preload) as the gold-standard and compared the ability of each device to detect this change across 3 temporal windows. Area under the receiver operator curve (AUROC) as well as the optimal threshold to detect altered preload at each temporal window from each device were calculated from Youden’s index. From this analysis, sensitivity and specificity were also calculated for each device at each temporal assessment window.

## Results

One subject was excluded because of diminished power from the wearable Doppler. Six subjects performed the protocol in triplicate while one subject performed the protocol in duplicate because of time-constraints in the physiology lab, thus 20 protocols were performed with a total of 40 preload changes. All subjects were male with an average age of 39. Average weight was 87 kg and BMI 26. On initiation of the protocols, the average HR was 76 beats per minute, systolic blood pressure 134 mmHg, diastolic blood pressure 79 mmHg and mean arterial pressure 99 mmHg. All hemodynamic variables were normal in distribution per the Kolmogorov-Smirnov test.

### Hemodynamic effects of ↑ preload

During the early hemodynamic window, heart rate did not change significantly, while mean arterial pressure increased by 8 mmHg (p = 0.01). SV from the non-invasive pulse contour device rose by 27 mL (+ 31%, p < 0.01). SV by bioreactance rose by 12 mL (+ 12%, p = 0.002). VTI and FTc from the wearable Doppler increased by 6 cm (+ 23%, p < 0.01) and 36 ms (+ 11%, p < 0.01), respectively.

During the middle hemodynamic window, heart rate fell significantly by 5 bpm (p = 0.004), while mean arterial pressure increased by 11 mmHg (p = 0.002). SV from the non-invasive pulse contour device rose by 24 mL (+ 28%, p < 0.01). SV by bioreactance rose by 17 mL (+ 17%, p = 0.001). VTI and FTc from the wearable Doppler increased by 7 cm (+ 29%, p < 0.01) and 35 ms (+ 11%, p < 0.01), respectively.

During the late hemodynamic window, neither heart rate nor mean arterial pressure changed significantly from baseline. SV from the non-invasive pulse contour remained elevated by 24 mL (+ 28%, p < 0.01). SV by bioreactance remained elevated by 17 mL (+ 17%, p = 0.001). VTI and FTc from the wearable Doppler increased by 5 cm (+ 23%, p < 0.01) and 38 ms (+ 12%, p < 0.01) from baseline, respectively.

### Hemodynamic effects of ↓ preload

During the early hemodynamic window, neither heart rate nor mean arterial pressure changed. SV from the non-invasive pulse contour device fell by 19 mL (- 17%, p = 0.0002). SV by bioreactance did not change significantly. VTI and FTc from the wearable Doppler decreased by 8 cm (- 26%, p < 0.01) and 34 ms (- 9%, p < 0.01), respectively.

During the middle hemodynamic window, neither heart nor mean arterial pressure changed significantly. SV from the non-invasive pulse contour device decreased by 23 mL (- 21%, p < 0.01). SV by bioreactance did not change significantly. VTI and FTc from the wearable Doppler decreased by 7 cm (- 22%, < 0.01) and 44 ms (- 12%, p < 0.01), respectively.

**Fig 2:**
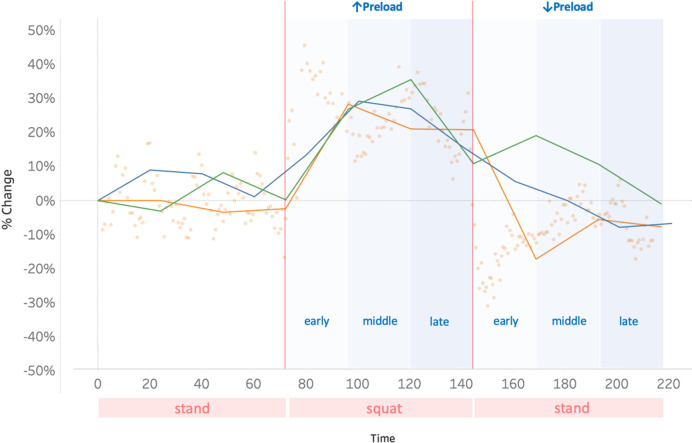
Representative protocol. Orange dots represent single beat VTI from the wearable carotid Doppler while the orange line depicts 24-second averages of the VTI. Green line is the 24-second average of SV from bioreactance. The blue line is a 20-second SV from non-invasive pulse contour. Y-axis is % change for each metric and x-axis is time.

During the late hemodynamic window, neither heart rate nor mean arterial pressure changed significantly from baseline. SV from the non-invasive pulse contour fell by 21 mL (-19%, p < 0.01). SV by bioreactance did not significantly change. VTI and FTc from the wearable Doppler fell by 6 cm (-19%, p < 0.01) and 39 ms (-11%, p < 0.01) from baseline, respectively. Fig 2 shows a representative summary of the data across time with the temporal windows highlighted for clarity.

### Temporal diagnostic characteristics of devices

**Fig 3:**
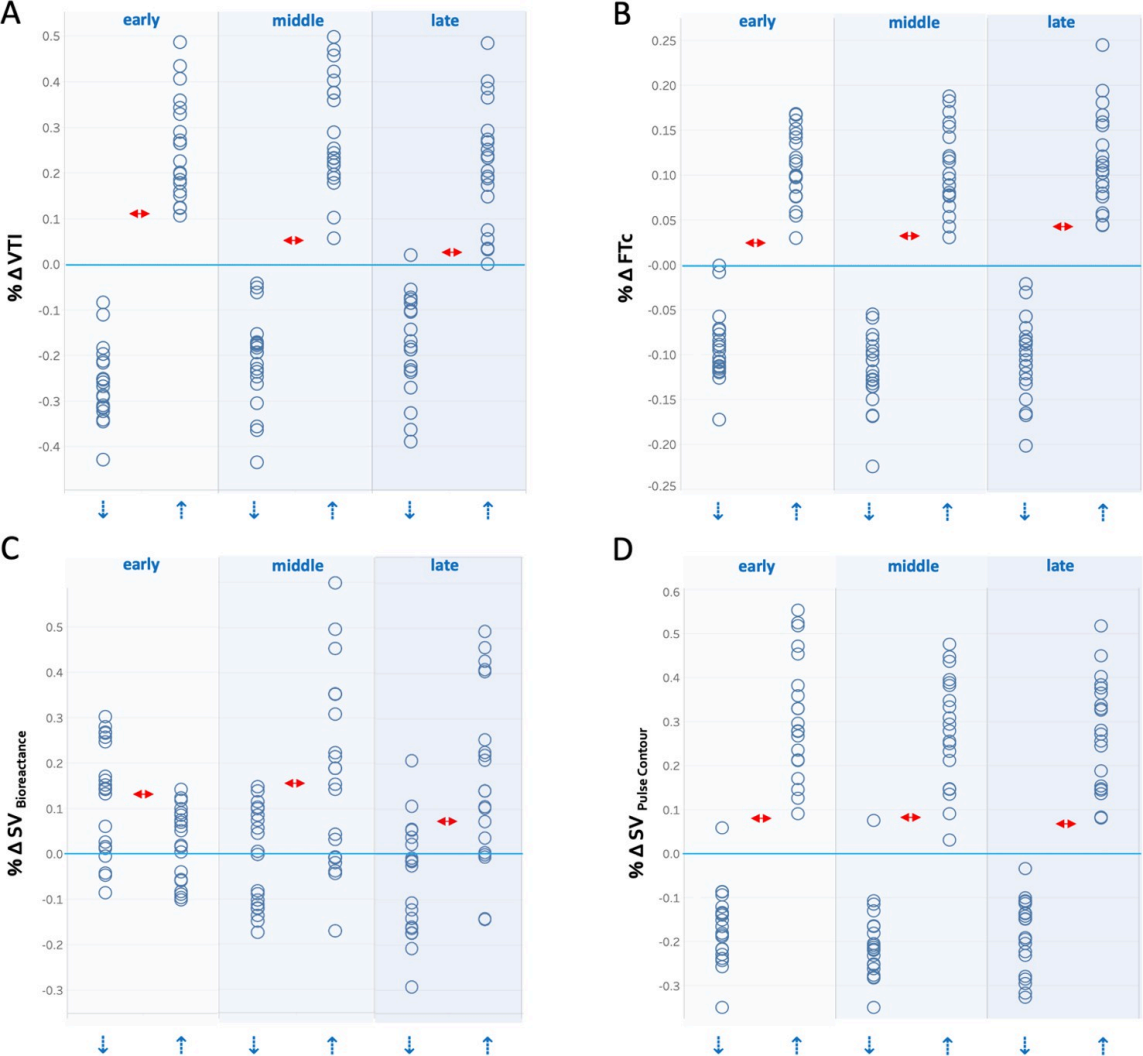
Dot-plots for each metric across all 3 temporal windows (early, middle, late). Horizontal, blue line is baseline for each measurement. The blue arrows represent directional change in preload. Y-axis is fractional change (% Δ) for each metric relative to baseline. A is velocity time integral (VTI) from the wearable Doppler, B. is corrected flow time (FTc) from the wearable Doppler, C. is stroke volume from bioreactance (SV_Bioreactance_) and D. is stroke volume from non-invasive pulse contour (SV_Pulse Contour_). The small, horizontal red arrows represent optimal diagnostic threshold for each metric. See Table 1 for threshold values.

A summary of the AUROC, optimal thresholds, sensitivity and specificities is shown in Table 1. Fig 3 shows the dot-plots for the metrics studied with thresholds superimposed.

**Table 1:** Diagnostic accuracy summary table.

	AUROC	Threshold	sensitivity	specificity
***early***				
**Carotid VTI**	0.98	10.5%	100%	100%
**Carotid FTc**	0.98	2.5%	100%	100%
**Bioreactance SV**	0.75	13%	60%	95%
**Pulse contour SV**	0.98	9%	100%	100%
***middle***				
**Carotid VTI**	0.98	5.5%	100%	95%
**Carotid FTc**	0.95	3%	100%	100%
**Bioreactance SV**	0.77	15%	55%	100%
**Pulse contour SV**	0.98	9%	100%	95%
***late***				
**Carotid VTI**	0.97	3%	95%	95%
**Carotid FTc**	0.98	4%	100%	100%
**Bioreactance SV**	0.85	7%	70%	90%
**Pulse contour SV**	0.98	8%	100%	100%

## Discussion

We observed that rapid preload changes induced by squatting and standing were more accurately detected, immediately, by a wearable Doppler and pulse contour analysis as compared to bioreactance. Importantly, the AUROC of bioreactance improved to 0.85 after 3 SV output intervals (i.e. 72 seconds). Implicit in this paradigm is that increased preload augments stroke volume and vice versa in all healthy volunteers. We believe that this is a correct assumption based on what is known about the physiology of squatting [14–16] and what we have previously reported in over 100 preload changes [9]. Squatting immediately removes the gravitational pressure gradient for venous return as the heart falls towards the feet, rapidly challenging the Starling mechanism. We note that a 50 mL crystalloid push over 10 seconds has been observed to increase stroke volume [17]; squatting mimics this physiology but more rapidly and reversibly.

We selected changing preload as the ‘gold-standard’ for two primary reasons. First, because of the rapid, reliable and reversible nature of squatting and standing, these maneuvers acted as well-defined physiological stresses to which the responses of 3 hemodynamic monitors could be compared. Second, because each device has inherent variation in its algorithm and ability to track change in left ventricular output, we could not define one specifically as a reference standard; indeed, a clinically-accepted ‘gold-standard’ for stroke volume remains ambiguous [18, 19]. Illustrating the difficulty in defining a true reference standard, our results resonate with a previous study in healthy volunteers where SV change measured by Doppler ultrasound better followed anticipated physiology than did bioreactance [20].

We believe that our observations are clinically-relevant. Notably, time-lag between physiological stress and the recorded-response by a gold-standard will degrade the sensitivity and specificity of a comparison device. In this paradigm, both carotid Doppler and non-invasive pulse contour response measured immediately after a rapid preload change would be considered discordant from bioreactance, when in actuality it may reflect algorithm-lag in the bioreactance device. For example, during increased preload, virtually all subjects increased carotid VTI and SV by pulse contour analysis by at least 10% during all three temporal windows (see Fig 3A, 3B and 3D). By contrast, the rise in bioreactance SV with increased preload stayed below 10% in a large fraction of subjects during the early assessment window and this gradually improved through the late window (see Fig 3C). The delayed response appeared even more prominent for bioreactance with diminished preload. As certain functional hemodynamic assessments demand rapid response times, that is, the ability to capture changes measured over time frames less than 5–10 seconds (e.g. the end-inspiratory/end-expiratory occlusion tests) and because hemodynamic changes *in vivo* can be very rapid [17], delayed output from a hemodynamic monitor might lead to erroneous clinical conclusions.

Our investigation has a number of limitations. First, it was a small sample size which was a function of convenience in the physiology lab where we worked. Thus, while our findings are applicable to healthy subjects, their reproducibility and accuracy have not been determined in critically-ill patients in whom the output of these devices are most germane. Because reliability and accuracy may differ due to dynamic changes in vascular tone, intra-thoracic pressure, ventricular compliance and myocardial geometry, additional studies are required before our findings are considered clinically-conclusive. Nevertheless, each participant performed multiple, rapid preload changes to increase our physiological sample size and studying healthy volunteers precludes the aforementioned pathologies from confounding the results. In other words, healthy volunteers in a physiology lab confer the optimal conditions for evaluating temporal concordance between devices. Second, the stand-squat-stand maneuver is not a well-known clinical maneuver as is, for example, passive leg raising. Yet, squatting and standing is easily performed with standardization and without additional equipment (e.g. a gurney for passive leg raise). Further, squat physiology is well-defined [8, 9] with previous investigations using both impedance cardiography and left ventricular outflow tract velocity time integral confirming the effects of squatting on stroke volume [14–16]. The benefit of this maneuver is that it introduced very rapid, specifically-defined preload changes from which we could track the temporal relationship of 3 monitors concurrently. Still, we did not directly assess change in venous return during squat and stand and, therefore, its change is inferred entirely by the changes in SV. We note that in a previous study using impedance cardiography–a precursor to bioreactance technology–both thoracic fluid volume and SV increased when squatting, which strongly implies enhanced venous return [16]. Additionally, echocardiography during squat revealed both increased left ventricular dimension and SV, also highly suggestive of enhanced venous return [15]. Further, the squat position when supine or when submerged in water has little cardiovascular effect, indicating that both kinking of the legs plays little role in squat hemodynamics and that the predominant mechanism is reduced gravitational pressure gradient between the heart and feet, as briefly mentioned above [14]. Third, without a defined SV gold-standard, we cannot be certain of the true left ventricular stroke volume change following rapid preload alteration. Yet, as previously reported, one subject performed the same stand-squat-stand maneuver while simultaneously measuring descending aortic VTI [11]; both squat and return-to-stand rapidly increased and decreased aortic VTI, respectively, consistent with the pattern noted in the common carotid artery. As well, because corrected systolic flow time (FTc) of the common carotid artery has been used as a successful, clinical surrogate for SV, and because FTc matched the carotid VTI response, our supposition that carotid Doppler followed SV in this paradigm is strengthened. Finally, because the wearable Doppler gives beat-to-beat data, we were able to average its temporal window to 24-seconds, that is, exactly match the bioreactance device. However, the pulse contour analysis gave outputs every 20-seconds, thus we could not exactly time-match this device. We phase-shifted the SV outputs for the pulse contour analysis and found no appreciable difference in sensitivity, specificity or AUROC for any temporal window studied.

## Conclusion

In conclusion, in a small sample of healthy volunteers, a wearable Doppler ultrasound and non-invasive pulse contour were better at rapidly-detecting preload change than bioreactance. The diagnostic accuracy of bioreactance improved and converged with the other monitors with time. Transient, temporal discordance between devices may represent ‘algorithm-lag’ and should be considered when interpreting clinical device comparisons. Functional hemodynamic assessments predicated upon rapid preload change and response measurement (e.g. end-expiratory occlusion) may be inaccurate as a function of temporal discordance. Further clinical investigation is needed.

## Supporting information

S1 Dataset (XLSX)
